# Influenza Infection in Mice Induces Accumulation of Lung Mast Cells through the Recruitment and Maturation of Mast Cell Progenitors

**DOI:** 10.3389/fimmu.2017.00310

**Published:** 2017-03-22

**Authors:** Behdad Zarnegar, Erika Mendez-Enriquez, Annika Westin, Cecilia Söderberg, Joakim S. Dahlin, Kjell-Olov Grönvik, Jenny Hallgren

**Affiliations:** ^1^Department of Medical Biochemistry and Microbiology, BMC, Uppsala University, Uppsala, Sweden; ^2^Uppsala Immunobiology Lab, National Veterinary Institute, Uppsala, Sweden

**Keywords:** mast cells, mast cell progenitors, recruitment, influenza, virus, lung

## Abstract

Mast cells (MCs) are powerful immune cells that mature in the peripheral tissues from bone marrow (BM)-derived mast cell progenitors (MCp). Accumulation of MCs in lung compartments where they are normally absent is thought to enhance symptoms in asthma. The enrichment of lung MCs is also observed in mice subjected to models of allergic airway inflammation. However, whether other types of lung inflammation trigger increased number of MCp, which give rise to MCs, is unknown. Here, mouse-adapted H1N1 influenza A was used as a model of respiratory virus infection. Intranasal administration of the virus induced expression of VCAM-1 on the lung vascular endothelium and an extensive increase in integrin β7^hi^ lung MCp. Experiments were performed to distinguish whether the influenza-induced increase in the number of lung MCp was triggered mainly by recruitment or *in situ* cell proliferation. A similar proportion of lung MCp from influenza-infected and PBS control mice were found to be in a proliferative state. Furthermore, BM chimeric mice were used in which the possibility of influenza-induced *in situ* cell proliferation of host MCp was prevented. Influenza infection in the chimeric mice induced a similar number of lung MCp as in normal mice. These experiments demonstrated that recruitment of MCp to the lung is the major mechanism behind the influenza-induced increase in lung MCp. Fifteen days post-infection, the influenza infection had elicited an immature MC population expressing intermediate levels of integrin β7, which was absent in controls. At the same time point, an increased number of toluidine blue^+^ MCs was detected in the upper central airways. When the inflammation was resolved, the MCs that accumulated in the lung upon influenza infection were gradually lost. In summary, our study reveals that influenza infection induces a transient accumulation of lung MCs through the recruitment and maturation of MCp. We speculate that temporary augmented numbers of lung MCs are a cause behind virus-induced exacerbations of MC-related lung diseases such as asthma.

## Introduction

Mast cells (MCs) develop from committed mast cell progenitors (MCp) in the bone marrow (BM), which enter tissues *via* the blood and mature into MCs (1). These cells play a crucial role in life-threatening allergic reactions such as in anaphylaxis and asthma attacks. In patients with asthma, MCs accumulate in the airway smooth muscles and lung epithelium (2, 3). The increase in MC numbers, particularly at these places in the lung, likely worsens the symptoms of the disease. Respiratory virus infections are the major cause of exacerbations of asthma (4). The exacerbations lead to suffering for the patients, and in worse case, they can have a fatal outcome.

Influenza infection is one of the most common respiratory virus infections associated with acute asthma exacerbations. This was especially studied during influenza A H1N1 worldwide outbreak in 2009, when asthma was one of the most common underlying medical conditions among hospitalized patients (5). MCs may play a role in influenza infections through their activation by pattern recognition receptors (6). In fact, mice lacking MCs (B6.Cg-*kit^W-sh^*) are protected from excessive inflammation following influenza A infection (7). Moreover, Graham et al. demonstrated that MCs can be activated by influenza virus *in vitro* to produce cytokines and that this process was dependent on activation of the pattern recognition receptor RIG-I. Therefore, MCs may contribute to the pathology associated with influenza infections.

We have previously studied the mechanisms behind the massive recruitment of MCp to the lung, which occurs in an experimental asthma model in mice (8–12). The influx of MCp to the lung, which is dependent on VCAM-1 on the lung vascular endothelium and the expression of α4-integrins on the MCp (8), was followed by an increase in mature MCs in the lung (9, 12, 13). Interestingly, VCAM-1 transcripts in the lungs of mice are upregulated from 2 to 8 days after influenza infection (14). This indicates that MCp may be recruited in a VCAM-1-dependent manner upon influenza infection. Thus, we hypothesized that influenza infection can amplify the number of lung MCs through the accumulation of MCp. These MC lineage-committed progenitors in adult mice were originally identified in the BM (15, 16) and intestine (17). However, we demonstrated that MCp can be detected in mouse blood as lineage^−^ (Lin^−^) c-kit^hi^ T1/ST2^+^ integrin β7^hi^ CD16/32^hi^ cells (18). The majority of the blood MCp in the BALB/c strain express FcεRI (66%), whereas only a minority (25%) of blood MCp in the C57BL/6 strain are positive for this marker (18). In the periphery such as in the peritoneum and lungs, virtually all of the MCp express FcεRI (19). Hence, in the lungs of naïve mice, there are two MC populations that can be detected by flow cytometry, mature MCs with high side scatter (SSC) properties which lack or have low expression of integrin β7 and the MCp population that express high levels of integrin β7 and have lower SSC properties (19). Similarly, the maturity of lung MCs can be distinguished by SSC and expression level of integrin β7 in mice subjected to allergic pulmonary inflammation (13).

In this study, we tested whether influenza infection in mice could stimulate an increase in lung MCp. A laboratory virus strain, the H1N1 influenza A/PR/8/34 virus (PR8), was used. Since an enhanced number of MCp in the lung after influenza infection can be a result of either induced recruitment or *in situ* proliferation, several types of experiments were performed to distinguish between these mechanisms. Intranasal administration of PR8 induced recruitment of highly proliferating MCp to the lung. Fifteen days post-infection, while MCp were still the most frequent MC type in the lung, an immature MC population expressing intermediate levels of integrin β7 was detected by flow cytometry. At this time point, influenza-induced toluidine blue^+^ MCs were found in association with the inflammatory cells surrounding bronchioles (Br) in the upper central airways. They also frequently appeared in the perivascular region and even in or close to endothelial and epithelial cells. When the lung inflammation was resolved, the majority of the MCs that appeared after influenza infection were gradually lost, likely due to homeostatic mechanisms. Therefore, we conclude that influenza infection in mice induces a transient accumulation of MCs in the lung, which mainly occur through recruitment and maturation of MCp.

## Materials and Methods

### Animals

Age- and sex-matched mice were bred and housed in the animal facility at the National Veterinary Institute (SVA), Uppsala, Sweden. All mice were at least 6 weeks old when they were used for experiments. Wild-type BALB/c mice were originally obtained from Bommice (Ry, Denmark). CD45.1 congenic mice on BALB/c background were purchased from Jackson Laboratory (Bar Harbor, ME, USA) and subsequently bred in-house. This study was carried out in accordance with the recommendations of Jordbruksverket. The protocol was approved by Uppsala Djurförsöksetiska nämnd, Stockholms Norra Djurförsöksetiska nämnd, or Stockholms Djurförsöksetiska nämnd.

### Influenza A Virus Infection Protocol

Isoflurane-anesthetized mice were inoculated intranasally with 3–5 × 10^4^ TCID_50_ of the H1N1 influenza A/Puerto Rico/8/34 strain or given an equal volume of PBS. Influenza virus was propagated in embryonated hen’s eggs, and strain purity was confirmed by PCR as previously described (20). Mice were weighted just before infection or PBS treatment (day 0). Weight loss was monitored on all mice during the course of the experiment. Mice that lost ≥15% of their initial body weight (weight at day 0) before the planned termination day were euthanized and excluded from the study.

### Extraction of Lung Cells

**Figure 1:**
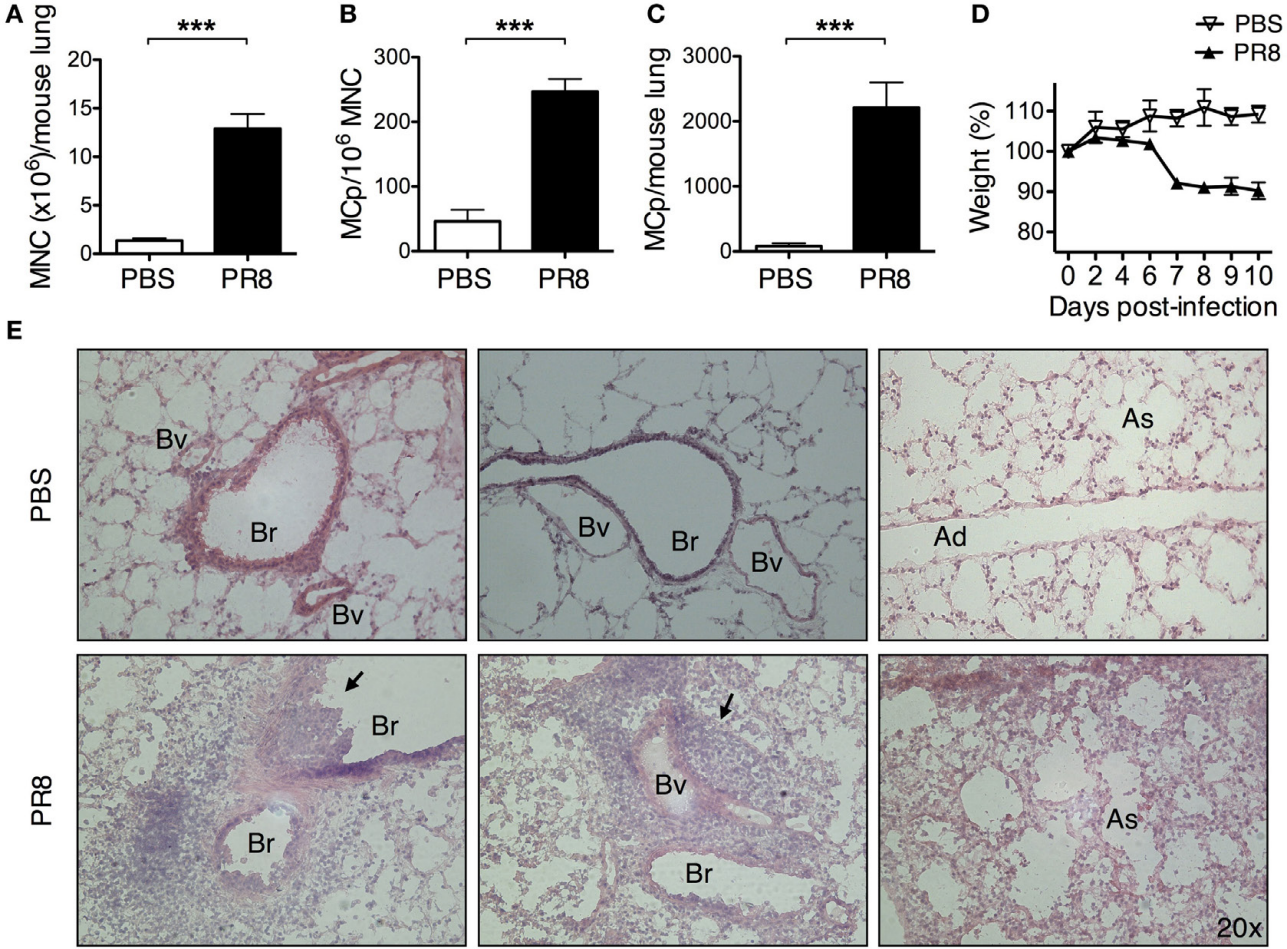
**Infection with PR8 influenza A virus increases the number of lung MCp**. Wild-type mice were infected with 3–5 × 10^4^ TCID_50_ PR8 influenza virus or instilled with PBS intranasally. Ten days post-infection, **(A)** lung mononuclear cells (MNC) were isolated and quantified by manual counting on a hemocytometer and **(B)** the frequency of MCp/10^6^ MNC and **(C)** the total number of MCp per mouse lung were determined using a limiting dilution and clonal expansion assay. The graphs in panels **(A–C)** show 6–10 individual mice per group pooled from 3 independent experiments. **(D)** The body weight was monitored during the course of the experiment. The data are normalized to each mouse’s initial body weight (weight at day 0). **(A–D)** All data are presented as mean ± SEM. **(E)** Representative pictures from H&E-stained lung tissue sections from PBS controls or PR8-infected mice 10 days post-infection. The black arrows point to the inflammatory cells that extravasated into the airway lumen or were associated with blood vessels (Bv). The inflammatory cells also frequently surrounded the bronchioles (Br). Ad, alveolar ducts; As, alveolar sacs (***P < 0.001).

The blood was removed from the lungs by injection of 10 ml PBS into the right ventricle of the heart. For results shown in Figure 1, lungs were minced with scalpels followed by enzymatic degradation of tissue by collagenase type IV (150 U/ml) (Life Technologies, Paisley, Scotland, UK) as previously described (12). In all other experiments, the lungs were mechanically and enzymatically dissociated into single-cell suspensions using the gentleMACS Octo Dissociator and mouse lung dissociation kit (Miltenyi Biotec, Bergisch Gladbach, Germany) according to the manufacturer’s instructions. The released lung cells were either enriched for mononuclear cells (MNC) using Percoll (Sigma-Aldrich, St. Louis, MO, USA) gradient centrifugation as described previously (12) or resuspended in 44% Percoll to remove the tissue residues and subsequently treated with red blood cell lysis buffer (150 mM NH_4_Cl, 9.5 mM NaHCO_3_, 1.2 mM EDTA). After purification, the viable cells were counted on a hemocytometer using trypan blue exclusion.

### Quantification of Lung MC Populations

A limiting dilution and clonal expansion assay (21) was carried out to quantify lung MCp in Figure 1. Briefly, lung MNC were cultured at eight different cell concentrations with IL-3, stem cell factor (SCF), and feeder cells in 96-well plates. After 12–14 days, the wells were scored as positive or negative for MC colony growth. The frequency of MCp/MNC was estimated using the Poisson distribution.

In the remaining figures, the frequency of lung MCp and mature MCs were quantified by flow cytometry. Lung MNC or whole lung cells were stained with the following monoclonal antibodies: Alexa Flour 700-conjugated CD45 (30-F11), PE-Cy7-conjugated c-kit (2B8), PE-conjugated FcεRIα (MAR-1), FITC-conjugated integrin β7 (FIB504), Brilliant Violet 605-conjugated CD16/32 (2.4G2), PE-Cy5-conjugated lineage antibodies to CD3 (17A2), CD4 (GK1.5), CD8b (eBioH35-17.2), CD11b (M1/70), CD19 (ebio1D3), Gr-1 (RB6-8C5), B220 (RA3-6B2), TER-119 (TER-119), and Brilliant Violet 421-conjugated T1/ST2 (DIH9) or biotinylated T1/ST2 (DJ8) followed by incubation with streptavidin-APC. Fluorescence minus one (FMO) controls with the appropriate isotype antibody were used. For detection of isoform-specific CD45, isolated MNC were stained with Brilliant Violet 421-conjugated CD45.1 (A20) and Alexa Flour 700-conjugated CD45.2 (104) antibodies instead of the general CD45 antibody. FMO controls were used to gate CD45.1^+^ and CD45.2^+^ MCp populations. To determine the percentage of proliferating (Ki-67^+^) MCp, isolated lung MNC were first stained with antibodies directed to surface markers. After fixation and permeabilization using the Foxp3/transcription factor staining buffer set (eBioscience, San Diego, CA, USA), the cells were blocked with 2% normal mouse serum followed by staining with Ki-67-PE (B56). A FMO control with the appropriate isotype antibody was used. The MCp were identified as CD45^+^ Lin^−/lo^ c-kit^hi^ T1/ST2^+^ CD16/32^int^ integrin β7^hi^ cells. The antibodies were from BD Biosciences (Franklin Lakes, NJ, USA), eBioscience (Hatfield, UK), or MD Bioproducts (Zürich, Switzerland). Stained cells were analyzed on a LSR II, LSR Fortessa flow cytometer, or FACS aria III (BD Biosciences), and data analysis was performed using the FlowJo software (TreeStar Inc., Ashland, OR, USA). In all flow cytometry analyses, doublet cells and debris were excluded. The total cell numbers from the quantifications are presented as the cell number/mouse lung. Mouse lung refers to all five lung lobes from a single mouse.

### Detection of *In Situ* Proliferation Using BrdU

Seven days after intranasal delivery of PR8 or PBS, all mice were lightly anesthetized by isoflurane inhalation and administered 16 mg/ml BrdU (0.8 mg/mouse) intranasally. The next day, mice were euthanized, the blood was removed, and the lungs were collected. After digestion of the lung tissue as described earlier, the lung cells were resuspended in 44% Percoll to remove the tissue residues. Thereafter, the lung cells were treated with red blood cell lysis buffer, stained with fluorescently labeled antibodies, fixated, and permeabilized as described for Ki-67. After intracellular staining with Brilliant Violet 510-conjugated anti-BrdU antibodies (3D4, BD Biosciences), the cells were analyzed on the LSR Fortessa flow cytometer. A FMO control with the appropriate isotype antibody was used to determine the gate for the BrdU^+^ cells.

### Generation of BM Chimeras

Bone marrow cells were isolated from femur and tibia of CD45.1 mice by flushing the bones using PBS. The cells from whole BM were counted on a hemocytometer using trypan blue dye, demonstrating a high degree of viable cells. The BM cells were injected intravenously (15 × 10^6^ cells per mouse) into γ-irradiated (5 Gy) BALB/c (CD45.2) mice. Two days later, mice were infected with PR8 or given PBS intranasally.

### Histological and Immunofluorescence Analyses

Lungs were removed and filled with 1.5 ml of OCT embedding media (Sakura Finetek, Alphenaan den Riijn, The Netherlands) diluted 1:1 with PBS, covered with OCT embedding media, and directly frozen in liquid nitrogen. The tissue was stored at −80°C until the section process. Eight-micrometer sections were cut with a cryostat and mounted on Menzel-Gläser Superfrost slides (Thermo Scientific, Braunschweing, Germany), air dried, and stored at −20°C until staining. To analyze the cellular infiltrates from day 10 post-infection, tissue sections were stained with hematoxylin and eosin (Histolab Products AB, Gothenburg, Sweden). To quantify the number of MCs, sections were stained with toluidine blue 1% and counter-stained with hematoxylin. The number of toluidine blue^+^ cells per slide was quantified in a blinded fashion using 20× magnification. The central airways were identified as airways proximal to the trachea branch in the upper lobes and the small airways were those with a small diameter, associated with the presence of alveolar sacs and respiratory bronchioles.

For immunostaining, slides were fixed in chilled 100% acetone (−20°C). After washing in PBS (pH 7.8), the slides were blocked with 5% horse serum (Sigma-Aldrich) in PBS for 30 min. The slides were stained with LEAF Purified anti-mouse CD106/VCAM-1 (BioLegend, San Diego, CA, USA) 1.5 μg/ml in 5% horse serum for 1 h. After washing twice in PBS, the slides were incubated with goat anti-rat Alexa Flour 594 (Life Technologies) 1 μg/ml in 5% horse serum for 1 h. After washing twice in PBS, the slides were mounted in DAPI (prolong Gold Antifade Mountant with DAPI, Life Technologies). The immunostained slides were analyzed with a LSM 700 confocal microscope (Carl Zeiss, Thornwood, NY, USA). At least five images from each section were taken, using Zen 2009 software (Carl Zeiss), and analyzed with ImageJ software (http://rsb.info.nih.gov/ij/) (NIH, Bethesda, MD, USA). The intensity per field was calculated in the Alexa Flour 594 channel and was reported as positive signal per field (raw integrated density − mean raw intensity of background readings). The fluorescence of background readings was taken in three regions next to the positive area in each image, and the average was used for the subtractions.

### Statistical Analysis

**Figure 2:**
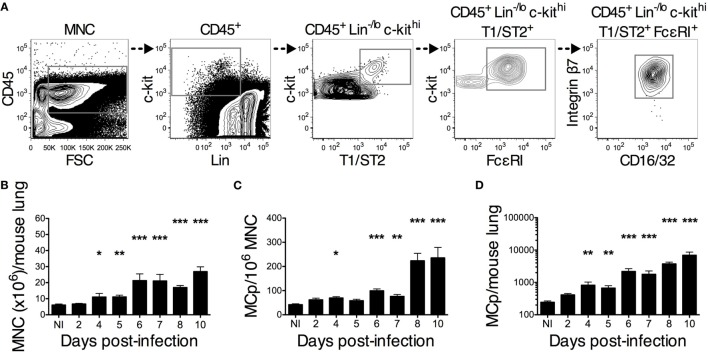
**PR8 infection stimulates an increase in lung MCp that starts on day 4 post-infection**. Wild-type mice were infected with 4 × 10^4^ TCID_50_ PR8 influenza virus and euthanized at the indicated days post-infection. Non-infected (NI) naïve mice were analyzed in parallel for comparison. **(A)** Representative gating strategy used for identification of lung MCp (the sample shown is from 10 days post-infection). MCp were identified as CD45^+^ Lin^−/lo^ c-kit^hi^ T1/ST2^+^ FcεRI^+^ CD16/32^int^ integrin β7^hi^ cells. **(B)** The number of mononuclear cells (MNC) per lung. **(C)** The frequency of the MCp was calculated by dividing the number of gated CD45^+^ Lin^−/lo^ c-kit^hi^ T1/ST2^+^ FcεRI^+^ CD16/32^int^ integrin β7^hi^ cells with the number of all MNC (pre-gated on singlet cells and debris excluded) and expressed as MCp/10^6^ MNC. **(D)** The total number of lung MCp was calculated for each mouse. The bars show the mean ± SEM of 6–14 individual mice per group pooled from 2 to 3 individual experiments, except for the NI, which is pooled data from 5 experiments in total (*P < 0.05, **P < 0.01, ***P < 0.001).

**Figure 3:**
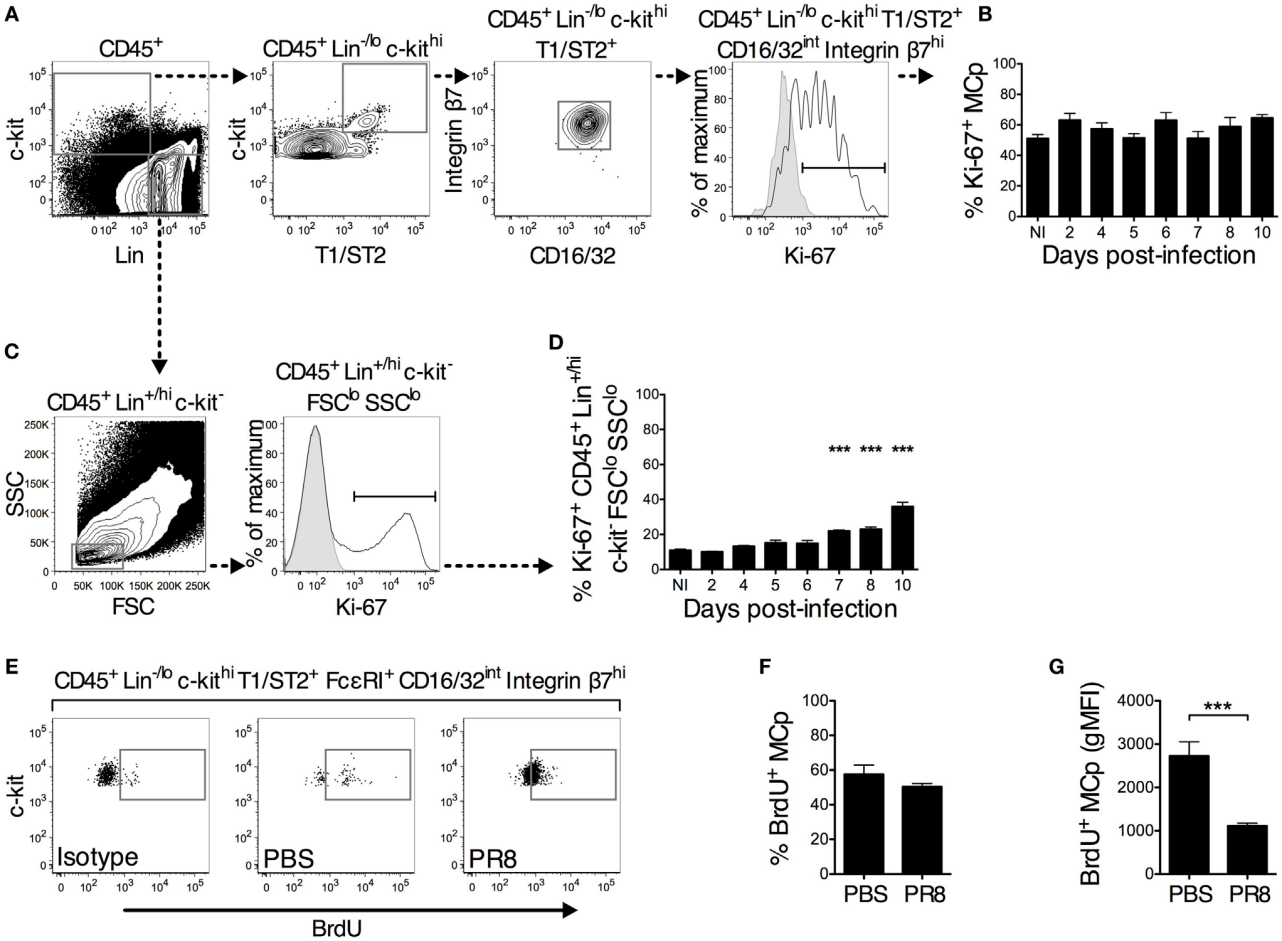
**A similar proportion of lung MCp from PR8-infected and naïve or PBS control mice are in a proliferative state**. **(A–D)** Wild-type mice were infected with PR8 influenza virus and euthanized at the indicated days post-infection. Non-infected (NI) naïve mice were analyzed in parallel for comparison. **(A,B)** The isolated lung mononuclear cells were stained with antibodies recognizing the indicated surface markers followed by intracellular staining with an antibody against Ki-67. **(A)** CD45^+^ Lin^−/lo^ c-kit^hi^ T1/ST2^+^ CD16/32^int^ integrin β7^hi^ lung MCp were analyzed for expression of Ki-67 following the illustrated gating strategy for a representative sample (the sample shown is from day 10 post-infection). **(B)** The percentage of Ki-67^+^ lung MCp in NI and PR8-infected mice at the indicated days post-infection. **(C)** CD45^+^ Lin^+/hi^ c-kit^−^ FSC^lo^ SSC^lo^ lung cells were analyzed for expression of Ki-67 following the illustrated gating strategy. **(D)** The percentage of Ki-67^+^ CD45^+^ Lin^+/hi^ c-kit^−^ FSC^lo^ SSC^lo^ lung cells in NI and PR8-infected mice at the indicated days post-infection. In panels **(A,C)**, the open white histogram shows cells stained with mouse anti-Ki-67 antibody, whereas the solid gray histogram shows a fluorescence minus one (FMO) control with an appropriate mouse IgG1 isotype antibody added. The graphs in panels **(B,D)** show the mean ± SEM of pooled data from 1–2 experiments for each individual day analyzed post-infection. The data represent 7–8 mice per group (days 5, 7, and 10 post-infection), 3–4 mice per group (days 2, 4, 6, and 8 post-infection), and 11 NI mice pooled from in total 6 experiments. **(E–G)** Seven days post-infection with PR8 influenza virus or installation of PBS, wild-type mice were given a single intranasal dose of BrdU. The next day, whole lung cells were analyzed for the presence of BrdU^+^ MCp. **(E)** Representative samples showing the gating for BrdU^+^ lung MCp, which was set using a FMO with the corresponding mouse IgG1 isotype control added. The mean ± SEM **(F)** percentage of BrdU^+^ lung MCp and **(G)** the geometric mean fluorescence intensity (gMFI) for the BrdU^+^ lung MCp from 9 mice per group pooled from 2 individual experiments are shown (***P < 0.001).

**Figure 4:**
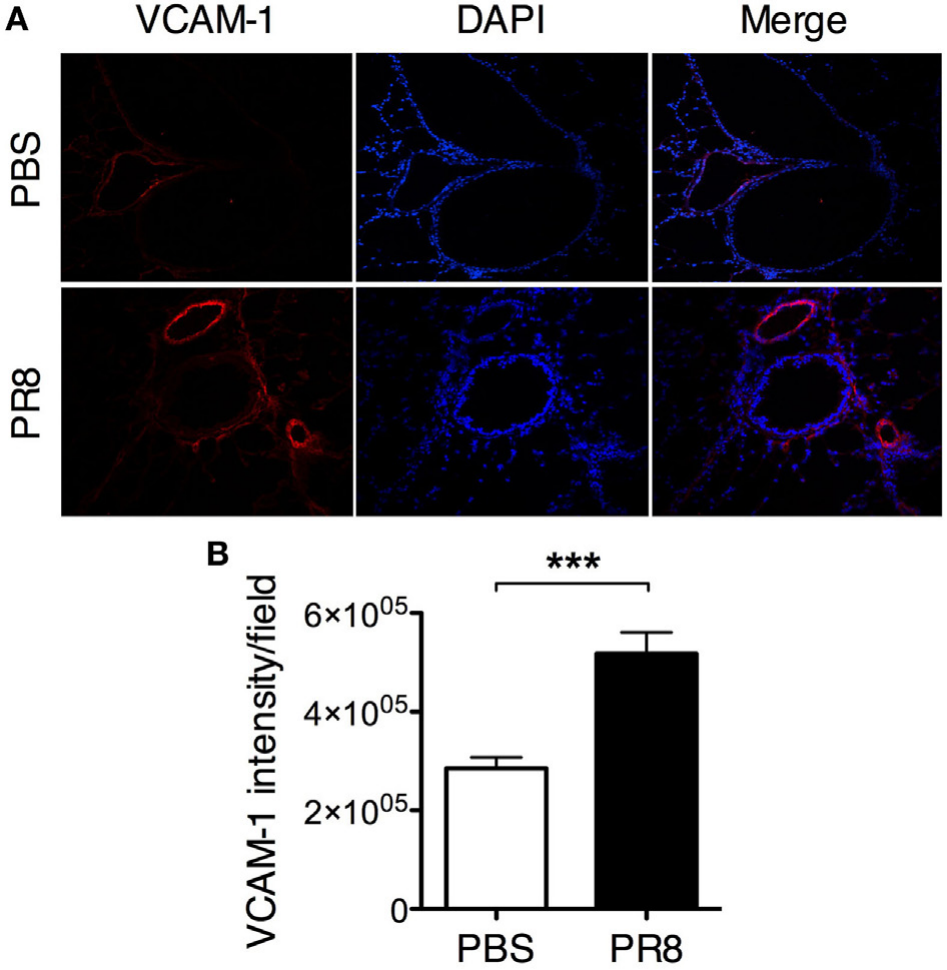
**PR8 infection induces upregulation of VCAM-1 expression on the lung vascular endothelium**. Lungs from PR8-infected and PBS control mice were collected 6 days post-infection. Sections of lung tissue were stained with rat anti-mouse VCAM-1 antibody (red) and DAPI (blue). **(A)** Representative immunofluorescence staining of mouse lung tissue, original magnification 10×. **(B)** Quantification of VCAM-1 expression in lung tissue sections. The graphs show the quantification of VCAM-1 intensity/field from 5 sections per mouse. The data are pooled from 2 independent experiments with 7–8 individual mice per group. The bars show mean ± SEM (***P < 0.001).

**Figure 5:**
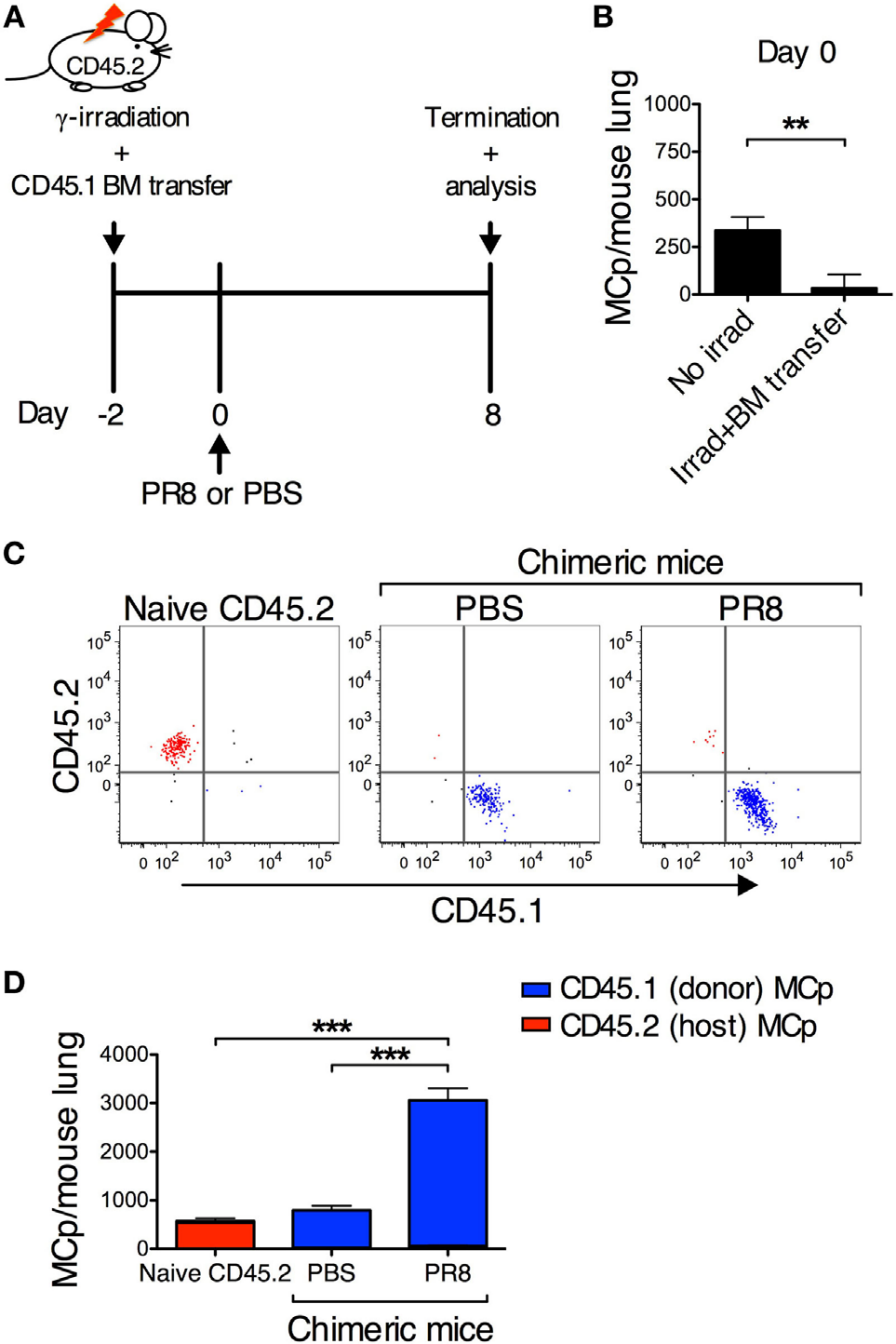
**PR8 infection induces the recruitment of MCp to the lung**. **(A)** CD45.2 BALB/c mice were sublethally γ-irradiated with 5 Gy and reconstituted with bone marrow (BM) cells from CD45.1 BALB/c mice, 2 days before infection (day 0). **(B)** The total number of lung MCp 2 days post γ-irradiation and BM transfer. The chimeric mice were either infected with PR8 or injected with PBS. Naïve non-irradiated CD45.2 mice were included as controls. **(C,D)** CD45.1^+^ MCp are represented in blue, while CD45.2^+^ MCp are represented in red. **(C)** Representative dot plots showing the CD45.1^+^ and CD45.2^+^ lung MCp populations. Fluorescence minus one controls were used to gate CD45.1^+^ and CD45.2^+^ MCp populations. **(D)** The total number of lung MCp 8 days post-infection. The graph in panel **(B)** show the mean ± SEM from 9–11 individual mice per group pooled from 4 independent experiments. The graph in panel **(D)** show the mean ± SEM from 12–15 individual mice per group pooled from 3 independent experiments (**P < 0.01, ***P < 0.001).

**Figure 6:**
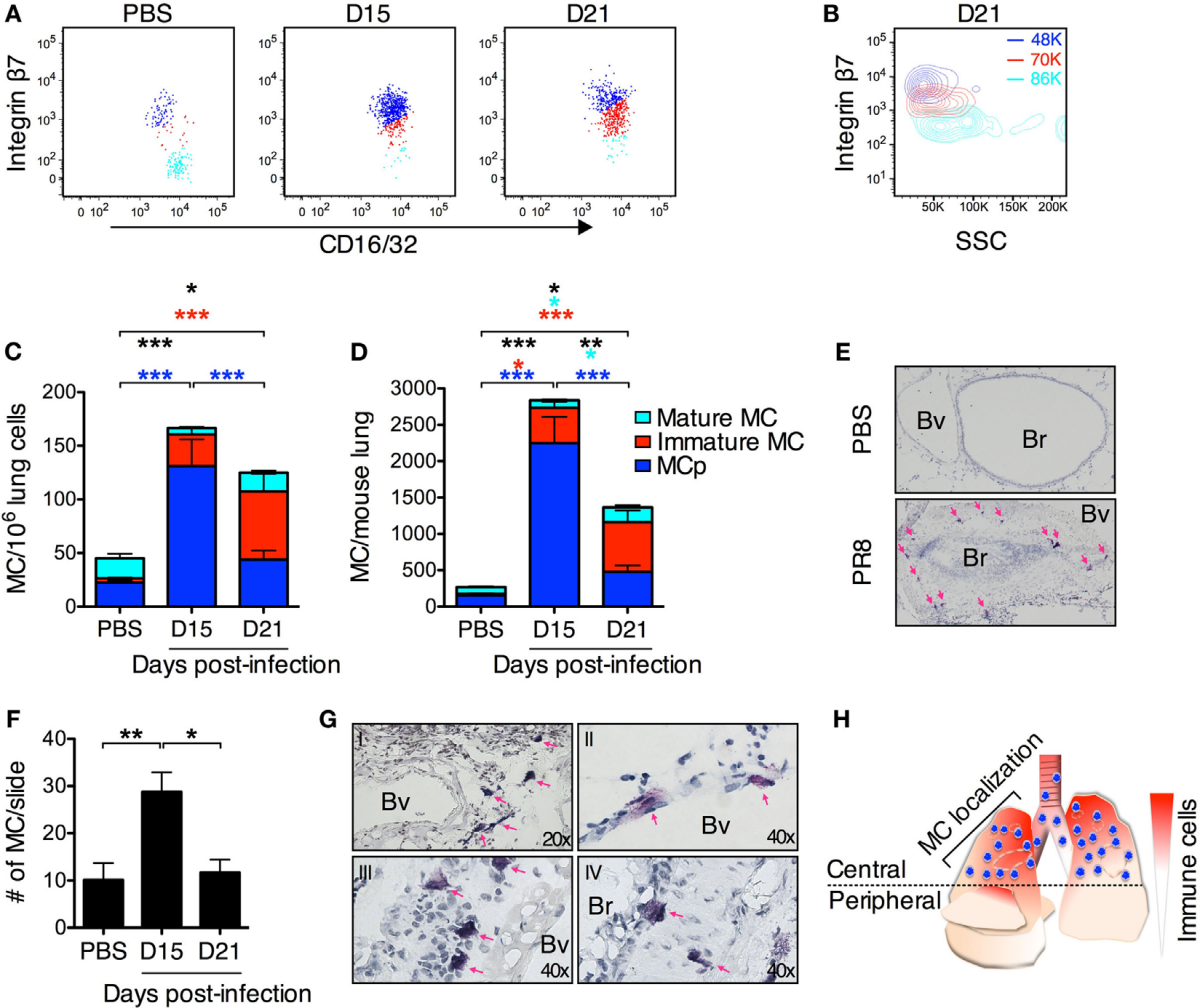
**PR8 infection induces a transient accumulation of lung MCs**. **(A)** Representative dot plots of lung mast cell populations from PR8-infected or PBS control mice analyzed at indicated time points post-infection. Three CD45^+^ Lin^−/lo^ c-kit^hi^ T1/ST2^+^ FcεRI^+^ populations are distinguished based on their integrin β7 surface expression in lungs from PR8-infected mice. The gates to identify the MCp and mature MCs were first set according to the two major MC populations detected in the lungs from PBS control mice in each individual experiment. Subsequently, a third gate was set for the MCs expressing intermediate levels of integrin β7 at each indicated time point after PR8 infection. **(A–D)** MCp (integrin β7^hi^; dark blue), immature MCs (integrin β7^int^; red), and mature MC (integrin β7^−/lo^; light blue). **(B)** Representative contour plot showing the side scatter (SSC) profile of the three different lung MC populations at day 21 post-infection. The numbers in color show the average of the mean SSC for each MC population from all experiments. **(C)** The frequency (MC/10^6^ lung cells) and **(D)** the total number of lung MC in PBS control mice and from PR8-infected mice at the indicated days post-infection. The proportion of each MC sub-population is distinguished by the corresponding color. The graphs in panels **(C,D)** show data from 8–12 individual mice per group pooled from 2 experiments at day 15, 3 experiments at day 21, and from all 5 experiments for PBS controls. Black stars show the statistical difference between groups for the sum of all lung MC populations. The light blue stars represent the level of significance for the mature MC population. The red stars represent the level of significance for the immature MC population. The dark blue stars represent the level of significance for MCp (*P < 0.05, **P < 0.01, ***P < 0.001). **(E)** Representative images of toluidine blue-stained sections from mice given PBS as a control and PR8-infected mice 15 days post-infection. The majority of MCs were found in the upper lobes in the central airways surrounding Bv and Br in the same region that contain other infiltrating immune cells. The pink arrows indicate MC. Br, bronchioles; Bv, blood vessels. **(F)** MC counts per lung section in the central airways. The graph represents pooled data from three independent experiments with 6–8 mice per group (*P < 0.05, **P < 0.01). **(G)** Representative images of toluidine blue-stained sections from PR8-infected mice 15 days post-infection divided into I–IV for illustrating where MCs are uniquely located after PR8 infection. (I) MCs were frequently found in the central airways in the perivascular region. (II) MCs were also found close to or within the endothelium. (III) MC in the sub-mucosal region. (IV) Intraepithelial and sub-mucosal MC. Pink arrows indicate MC. Br, bronchioles; Bv, blood vessels. **(H)** Graphical representation of the MC localization in the lung in PR8-infected mice day 15 post-infection. MCs are represented in blue and other cells/inflammation in red.

Statistical differences between groups were assessed using unpaired, two-tailed Student’s *t*-test for Figures 1, 3F,G, 4 and 5B. For Figures 2 and 3B,D, one-way ANOVA with Dunnett’s test for pairwise comparison was performed to determine the statistical significance. For Figure 2, the data were log-transformed to compensate for unequal variation between the control group and the treatment groups before performing the statistical analysis. In Figures 5D and 6, all groups were compared to each other, and therefore, the statistical significance was determined by one-way ANOVA with *post hoc* Tukey’s test. All graphs were prepared and statistics calculated using GraphPad Prism 5.0c (GraphPad software Inc., San Diego, CA, USA). A *p*-value of less than 0.05 was considered significant.

## Results

### Influenza Infection Stimulates a Massive Increase in the Number of Lung MCp

MCs accumulate in the lung of asthmatics and in mice subjected to experimental models of allergic pulmonary inflammation. In such models, the MC accumulation is preceded by recruitment of MCp to the lung (1). However, whether other types of stimuli of the lung such as influenza infection can trigger a similar type of response has remained unknown.

Wild-type BALB/c mice were given PBS or a sublethal dose of PR8 virus intranasally. This virus causes respiratory disease that can be monitored by weight loss. Ten days post-infection, the yield of lung MNC increased around 9 times in PR8-infected mice as compared with PBS-treated mice (Figure 1A). On the same day, the frequency of lung MCp was approximately 5 times higher in PR8-infected mice (Figure 1B), and the total number of lung MCp per mouse was around 28 times higher than in PBS control mice (Figure 1C), as evaluated by a limiting dilution and clonal expansion assay (21). Ten days post-infection, the PR8-infected mice had lost on average 10% of their initial body weight (weight at day 0) (Figure 1D). At this time point, inflammatory cells had accumulated around bronchioles and blood vessels in the lungs (Figure 1E, left and middle panels) and pneumonia could be detected (Figure 1E, right panel).

### Influenza Infection Induces a Time-Dependent Increase in Lung MCp

A flow cytometry approach was used to investigate the kinetics of the PR8-induced increase in lung MCp. Lung MCp were identified as CD45^+^ Lin^−/lo^ c-kit^hi^ T1/ST2^+^ FcεRI^+^ CD16/32^int^ integrin β7^hi^ cells, similar to what we previously described (19) (Figure 2A). The PR8-induced increase in lung MCp was followed over a course of 10 days post-infection. On average, a significant increase in MNC as well as in the frequency and total number of lung MCp per mouse was detected 4 days post-infection (Figures 2B–D). However, on days 4 and 5, there is a large variation in the frequency of MCp between the PR8-infected mice, i.e. some of the individual mice have a higher MCp frequency in the lung than PBS control mice, and some do not. From 6 days post-infection and beyond, there was a robust increase in the frequency and total number of lung MCp per mouse (Figures 2C,D). Remarkably, at day 10 post-infection, the total number of lung MCp per mouse was approximately 29 times higher than in naïve mice (Figure 2D). The PR8-induced fold increase in total lung MCp quantified by flow cytometry was at a similar level as the fold increase detected by the limiting dilution assay (compare Figure 2D to Figure 1C).

### A Similar Proportion of Lung MCp from PR8-Infected and Naïve or PBS Control Mice Are in a Proliferative State

The increase in the frequency and number of lung MCp after PR8 infection could theoretically be explained by virus-induced stimulation of MCp cell division or recruitment of MCp from the blood to the lung. To determine whether the PR8 infection affected the proliferation state of lung MCp, the proportion of Ki-67^+^ MCp was assessed following the gating strategy shown in Figure 3A. Ki-67 is an intracellular protein expressed only in proliferating cells (22, 23). A FMO control with an appropriate mouse IgG1 isotype antibody added was used to determine the Ki-67^+^ proportion of the cells. The proportion of proliferating Ki-67^+^ MCp among total lung MCp was approximately 51% in naïve mice (Figure 3B). PR8 infection did not increase the proliferative state of the MCp (Figure 3B). However, in 1 out of the 6 experiments, there was a significantly higher percentage of Ki-67^+^ lung MCp 10 days post-infection than in naïve mice when analyzed by a Student’s *t*-test (60 ± 2 vs. 45 ± 4%).

Lymphocytes such as activated CD8^+^ T cells continue to proliferate after their arrival to the lungs in influenza infection (24). Therefore, CD45^+^ Lin^+/hi^ c-kit^−^ forward scatter (FSC)^lo^ SSC^lo^ lung cells, which likely consists of mostly lymphocytes, were gated for their proportion of Ki-67^+^ cells (Figure 3C). In naïve mice, 11% of the cells in the CD45^+^ Lin^+/hi^ c-kit^−^ FSC^lo^ SSC^lo^ gate were Ki-67^+^. Nevertheless, 10 days post-infection, the proportion of Ki-67^+^ CD45^+^ Lin^+/hi^ c-kit^−^ FSC^lo^ SSC^lo^ cells had increased up to 36% (Figure 3D). Taken together, these results indicate that MCp from naïve mice are in a high proliferative state that remains constant after PR8 infection.

A BrdU incorporation assay was used to verify the conclusions from the Ki-67 staining of lung MCp (Figures 3E–G). On day 7 post-infection, a single intranasal installation of BrdU was given to mice infected with PR8 or treated with PBS. The following day, the percentage of BrdU^+^ MCp was determined (Figure 3E). A high percentage of lung MCp was BrdU^+^ in both groups of mice (Figures 3E,F). Following a similar gating strategy as in the lung, the CD45^+^ Lin^−/lo^ c-kit^hi^ T1/ST2^+^ FcεRI^+^ CD16/32^int^ integrin β7^hi^ MCp-like cells in the BM were also to a high degree BrdU^+^, suggesting that BrdU had diffused to the BM (Figure S1 in Supplementary Material). Although this prevents us from concluding that the BrdU positivity refers only to *in situ* cell division in the lung, these experiments clearly show that MCp in the BM and lung are dividing. The lung MCp from the PR8-infected mice had lower geometric mean fluorescence intensity (gMFI) for BrdU than the lung MCp from PBS control mice (Figure 3G), indicating that on average, each dividing MCp in the PBS control mice had taken up more BrdU than the BrdU^+^ MCp from PR8-infected mice. Collectively, the Ki-67 and BrdU data demonstrate that PR8 infection does not induce an increased cell proliferation of lung MCp.

### Influenza Infection Stimulates the Recruitment of MCp to the Lung

Upon allergic airway inflammation, MCp are recruited to the inflamed lung in a process that is dependent on the expression of α4β1- and α4β7-integrins on the MCp and induced expression of VCAM-1 in the lung vascular endothelium (8). Hence, the expression of VCAM-1 in the lung endothelium was compared between PR8-infected and PBS control mice. Indeed, PR8 infection induced VCAM-1 expression on the lung endothelium (Figures 4A,B). VCAM-1 expression was found on the veins, arteries, and postcapillary venules in PR8-infected mice. This suggests that MCp can be recruited from the blood to the lung during influenza infection. To test whether recruitment alone could account for the strong increase in lung MCp induced by PR8 infection, we took advantage of the CD45.1 strain, which is congenic to the wild-type BALB/c (CD45.2) strain. To generate BM chimeras, sublethally γ-irradiated CD45.2 BALB/c mice were reconstituted with BM cells from CD45.1 congenic mice (Figure 5A). We have previously shown that MCp are sensitive to γ-irradiation (9, 19). In line with this observation, lung MCp were found to be depleted 2 days after irradiation and BM transfer, i.e., day 0 (Figure 5B). The chimeric mice were infected with PR8 or given PBS as control at this time point. The lungs of both groups of chimeric mice were analyzed by flow cytometry day 8 post-infection using isoform-specific anti-CD45.1 and anti-CD45.2 antibodies (Figure S2A in Supplementary Material). As expected, the lung MCp from naïve wild-type mice were expressing the CD45.2 isoform and the vast majority of the lung MCp from PR8-infected or PBS-treated chimeras were CD45.1^+^ cells (Figure 5C). This shows that the lung MCp present at day 8 post-infection originate from the donor BM cells and that host MCp were still depleted. Thus, in this experimental system, the possibility of PR8-induced cell proliferation of host MCp *in situ* has been prevented. PR8-infected chimeras had a significantly higher number of lung MCp per mouse compared to naïve non-irradiated CD45.2 mice and PBS-treated control chimeras (Figure 5D). Importantly, the total number of lung MCp per mouse in chimeric mice 8 days post-infection were similar to the total number of lung MCp in normal non-irradiated mice at the same day post-infection (3,056 ± 264 in Figure 5D vs. 3,739 ± 489 in Figure 2D). This indicates that PR8 stimulates an influx of MCp to the lung, to a similar extent as in non-irradiated PR8-infected mice. Thus, the data strongly suggest that recruitment of MCp from the blood to the lung is the major mechanism behind the PR8-induced increase in lung MCp. Moreover, PR8-infected chimeras had a significantly higher number of lung MNC per mouse and frequency of lung MCp compared to naïve non-irradiated CD45.2 mice and PBS-treated control chimeras (Figures S2B,C in Supplementary Material).

Altogether, the PR8-induced upregulation of VCAM-1 expression on the lung vascular endothelium and the capacity of PR8 to stimulate a similar number of lung MCp in the chimeric mice and in normal non-irradiated mice strongly suggest that the major mechanism behind the influenza-induced increase in MCp numbers is recruitment of blood MCp to the lung.

### A Fraction of the Recruited MCp Develops into MC, but the Majority of These MCs Are Lost when the Influenza-Induced Inflammation Resolves

To address whether the MCp recruited to the lung upon influenza infection are maturing into MCs, a different lung cell isolation method to analyze all lung MC populations was set up. In these experiments, the cells released after mechanical and enzymatic digestion of whole lungs were only purified by removal of debris and lysis of red blood cells. Using flow cytometry, the CD45^+^ Lin^−/lo^ c-kit^hi^ T1/ST2^+^ FcεRI^+^ cells were gated for expression of integrin β7 and CD16/32 to analyze all forms of lung MCs. In mice given PBS as a control, two major CD45^+^ Lin^−/lo^ c-kit^hi^ T1/ST2^+^ FcεRI^+^ MC populations could be identified (Figure 6A). One cell population expressed high levels of integrin β7 and had a low mean SSC, which corresponds to the lung MCp population (Figures 6A,B, dark blue). The second cell population lacked or was expressing low levels of integrin β7 and had a high mean SSC profile, which are properties unique to the mature MC population in the lung (Figures 6A,B, light blue). However, at days 15 and 21 post-infection, three MC populations were identified. Besides the MCp and the mature MC population, a cell population with intermediate expression of integrin β7 could be found (Figure 6A, red). This immature MC population had a mean SSC that were in between the mature MCs and the lung MCp (Figure 6B). At day 15 post-infection, the MCp population was still the dominant MC type in the lung, whereas the immature MC population was the most frequent of the three MC types at day 21 post-infection (Figures 6C,D). Although there was a reduction in the total number of MCs per mouse lung day 21 compared with day 15 post-infection, the numbers were still higher than in the mice given PBS as a control (Figure 6D). The number of immature MCs was increased at day 21 as compared to day 15 post-infection, while the number of lung MCp per mouse was reduced (Figure 6D). In addition, there were more mature MCs in the lung day 21 than day 15 post-infection or in comparison to mice given PBS as a control (Figure 6D).

Histological analyses were performed to investigate in which locations the influenza-induced MCs accumulate. We found that both the PR8-induced inflammation and MC accumulation were mainly localized to the central upper airways (Figure 6H). This location is consistent with the localization of influenza infection, which begins in the large conducting airways later spreading to the bronchioles and deeper areas of the lung (25). The number of toluidine blue^+^ MCs in this area of the lungs were quantified by manual counting under the microscope in a blinded fashion. In mice given PBS, there was no inflammatory cell infiltrates around bronchioles or blood vessels (Figure 6E). However, 15 days post-infection with PR8, most Br in the upper airways were surrounded with inflammatory cells and often these inflamed bronchioles contained toluidine blue^+^ MCs (Figure 6E). In fact, there were significantly more toluidine blue^+^ MCs in these locations in the lung at 15 days post-infection than in control mice given PBS (Figure 6F). At this time point, interstitial MCs in the perivascular space (Figure 6G, panel I), in the endothelium (II) or just under the endothelium (III) and intra- or sub-epithelial MCs (IV) were also detected in the PR8-infected lungs. Interestingly, although the number of lung MCs was still significantly higher in PR8-infected mice than in PBS-treated mice when analyzed by flow cytometry day 21 post-infection (Figure 6D), histological analysis demonstrated similar numbers of toluidine blue^+^ MCs in the upper airways of PR8-infected and PBS control mice (Figure 6F).

Altogether, these data suggest that PR8 infection induces MC accumulation in the lung, which is present as long as the inflammation is ongoing. However, most MCs that originate from recruited MCp are lost when the inflammation is resolved.

## Discussion

Although earlier studies have shown that MCp are recruited to the lung and give rise to MCs in mouse models of allergic airway inflammation (8, 9, 12, 13), the present study demonstrates for the first time that MCp are recruited to the lung during a completely different type of inflammatory response, i.e., influenza infection. The H1N1 influenza A virus triggered a tremendous increase in highly proliferating lung MCp, which was significant already 4 days post-infection and continued to increase over time during the acute phase. However, as lung leukocytes contain a significant proportion of intravascular cells that cannot be removed by perfusion of the lungs (26), some of the lung MCp that we quantify may be trapped in the lung vasculature.

Fifteen days post-infection, the majority of the CD45^+^ Lin^−/lo^ c-kit^hi^ T1/ST2^+^ FcεRI^+^ cells in the lung were still MCp. At this time point, an immature MC population that expressed an intermediate level of integrin β7 and had an intermediate SSC profile was also detected. In line with this finding, a similar transitional MC population was demonstrated before the appearance of induced mature MCs in a model of allergic pulmonary inflammation (13). Therefore, both influenza infection and allergic inflammation trigger a strong influx of MCp to the lung and stimulate the maturation of MCp *via* a similar transitional maturation step. However, in our study, the mature MC population, expressing none or low levels of integrin β7, was equally rare as in PBS control mice 15 days post-infection. At this time point, increased numbers of toluidine blue^+^ MCs could be found close to the inflamed bronchioles in the upper and central airways. Toluidine blue^+^ lung MCs could also be detected in perivascular spaces and close to or in the endothelial or epithelial barriers in the PR8-infected mice. MCs are rarely detected among the inflammatory foci in mouse models of allergic airway inflammation but instead accumulate in the lung epithelium (9, 27), close to the airway smooth muscles (28) and in the alveolar parenchyma (12). Hence, our data suggest that the influenza infection in mice triggers lung MCs to accumulate at partly different locations than in allergic airway inflammation.

The increase in lung MCp upon influenza infection could theoretically be due to virus-induced increase in cell proliferation of lung MCp *in situ*, or to the recruitment of MCp to the lung *via* the blood. Our data suggest that lung MCp from naïve and influenza-infected lungs are in an equally high proliferative state because more than half of the MCp were positive for the cell proliferation marker Ki-67 or had incorporated BrdU. The high proliferative state of the rare MCp in naive mice is likely required to sustain the replacement of the constitutive MC pool in the lung. Interestingly, the BrdU^+^ lung MCp from the PR8-infected mice had a lower gMFI than those from mice given PBS. We speculate that the higher gMFI in the PBS control mice reflects that most (if not all) lung MCp were resident at the time point when BrdU was given, whereas in the PR8-infected mice, many MCp were recruited between days 7 and 8, and therefore, the gMFI of BrdU^+^ lung MCp is lower. Nevertheless, the equally high proportion of Ki-67^+^ or BrdU^+^ lung MCp in naïve and PR8-infected mice implies that recruitment would be the major mechanism behind the influenza-induced increase in the number of lung MCp. To test this, CD45.2 mice depleted of lung MCp and reconstituted with BM cells from congenic CD45.1 donors were instilled intranasally with PBS or infected with PR8. This experimental system allowed us to test whether the strong increase in lung MCp induced by influenza infection could be due to recruitment alone since the host lung MCp were depleted from the lung before infection and remained so until the day of analysis. The PR8 infection induced a stronger influx of MCp to the lung than the PBS treatment alone in the chimeric mice. Importantly, the high number of lung MCp (of donor origin) in the PR8-infected chimeric mice was similar to the total number of lung MCp induced by PR8 infection in normal mice. Therefore, these experiments clearly show that the increase in MCp upon PR8 infection could be explained entirely by the recruitment of MCp from the blood to the lung. However, the results from the irradiation and transfer experiments need to be interpreted with caution since injection of whole BM cells into the blood circulation leads to an artificial presence of all types of hematopoietic progenitor cells in the circulation, and the irradiation may cause increased vascular permeability in the lung (29, 30). Importantly, PR8 infection significantly enhanced the expression of VCAM-1 on the lung endothelium. Since we previously showed that MCp were recruited to the lung in a VCAM-1-dependent fashion in a mouse model of allergic airway inflammation (8), the induced VCAM-1 expression suggests that MCp use the same transmigration mechanism in influenza infection and allergic airway inflammation. Altogether, we conclude that influenza infection triggers the recruitment of MCp, which are in a high proliferative state, to the lung.

Three weeks post-influenza infection when most of the lung inflammation was resolved, the total sum of the individual lung MC populations was reduced by 50% compared to day 15, although the total sum of MCs in different developmental stages were still 4 times higher than in PBS-treated mice. This indicates that homeostatic mechanisms also apply to the influenza-induced MC populations. At the same time, the number of toluidine blue^+^ cells in the upper airways was similar to the basal levels detected in mice given PBS. Although this may seem inconsistent, it is easier to detect differences when MCs are quantified by flow cytometry than by histological analysis. Moreover, the histological quantifications were performed on the central and upper small airways where both the inflammation and the MC accumulation were focused, whereas the flow cytometry was performed on cells from homogenized whole lungs. Indeed, also in mice subjected to an experimental asthma model, the majority of the MCs are localized to the central airways (31). Hence, the residual influenza-induced MCs detected by flow cytometry at day 21 post-infection may be dispersed throughout the lungs, and therefore, a difference would not be easily detectable by histology. We speculate that the reduction of the influenza-induced MCs takes place by Fas-mediated cell death accompanied with local phagocytic clearance as described for influenza-induced recruited macrophages during the resolution phase of PR8 infection (32). Another plausible explanation is that there are too low levels of SCF present after the resolution of PR8-induced inflammation to sustain the long-term survival of the lung MCs which originate from recruited MCp. SCF is the only known crucial factor that is truly necessary for survival of tissue MCs *in vivo*. This is perhaps best exemplified by different MC-deficient mouse strains, which lack MCs due to loss of a functional receptor for SCF, Kit, e.g., W/Wv or Wsh/Wsh mice, or lack SCF (Kitl^Sl^/Kit^Sl-d^ mice) (33–35). Therefore, the PR8-induced MCs may die through Bim-mediated growth factor deprivation-induced apoptosis (36). However, determining the precise mechanism behind the loss of influenza-induced MC accumulation in the lungs was beyond the scope of this investigation.

The present study demonstrates the accumulation of MCs in the airways in response to a respiratory virus infection in mice. However, in other species, increases in MC numbers have been observed previously in connection with different types of respiratory virus infections. For example, an increased MC number was demonstrated in bronchoalveolar lavage fluid and in the airway lumen of dogs infected with influenza C virus (37). In rats, parainfluenza type 1 virus (Sendai) induced an increase in the number of bronchiolar MCs (38). In addition, humans naturally infected with cold had increased number of MCs in the sub-epithelial layer of the nasal mucosa (39). Thus, the induction of increased number of MCs in affected tissues upon respiratory virus infections seems to be a general phenomenon and not restricted to mice or the PR8 influenza strain.

In human asthma, MC numbers are increased in certain locations of the lung, i.e., in the airway smooth muscles and lung epithelium (2, 3). However, also the phenotype of MCs present in human asthmatics differs dependent on severity. For example, in severe asthmatics, MCs of the tryptase^+^/chymase^+^ phenotype are most predominant in the airway submucosa and epithelium (40). We recently identified a rare human MCp population in blood that, similar to its counterpart in murine lung, expresses FcεRI and integrin β7 (41). These human MCp are likely the immediate precursors to human tissue MCs. Therefore, accumulation of lung MCs due to recruitment and maturation of MCp in response to virus infections may occur in humans as well.

MCs may play a role in initiating or amplifying the immune reactions when activated through pattern recognition receptors during influenza infection (6). For example, both human and mouse MCs derived *in vitro* become activated in a RIG-I-dependent fashion upon influenza infection (7). However, since MCs presumably cannot directly eliminate viruses or virus-infected cells, the participation of MCs for making a protective host defense against respiratory virus infections is likely limited. Instead, MCs may contribute to an excess pathology and deteriorating lung function in certain individuals through their activation and release of mediators. Altogether, our data in mice show that the recruitment of MCp to the lung during influenza infections will increase the MC burden at least temporarily over a few weeks. We speculate that the accumulation of MCs due to respiratory virus infections contributes to the increased symptoms seen in patients with asthma that suffer from virus-induced exacerbations. In such occasions, MCs and possibly also MCp may be activated by IgE/antigen and/or *via* pattern recognition receptors. Moreover, asthma patients, due to the chronic inflammation in their lung, may have sustained production of survival factors such as SCF and may thus retain virus-induced accumulations of MCs for a longer time.

## Author Contributions

BZ, EM-E, and AW performed the experiments. CS, JSD, and K-OG contributed to the initial experiments. JH initiated the project. BZ, EM-E, and JH designed the experiments, interpreted the data, and wrote the manuscript.

## Conflict of Interest Statement

The authors declare that the research was conducted in the absence of any commercial or financial relationships that could be construed as a potential conflict of interest. The reviewer SB and handling editor declared their shared affiliation, and the handling editor states that the process nevertheless met the standards of a fair and objective review.

## References

[1] Dahlin JS Hallgren J. Mast cell progenitors: origin, development and migration to tissues. Mol Immunol (2015) 63:9–17. 10.1016/j.molimm.2014.01.018 24598075

[2] Brightling CE Bradding P Symon FA Holgate ST Wardlaw AJ Pavord ID Mast-cell infiltration of airway smooth muscle in asthma. N Engl J Med (2002) 346:1699–705. 10.1056/NEJMoa012705 12037149

[3] Dougherty RH Sidhu SS Raman K Solon M Solberg OD Caughey GH Accumulation of intraepithelial mast cells with a unique protease phenotype in T(H)2-high asthma. J Allergy Clin Immunol (2010) 125:1046–53.e8. 10.1016/j.jaci.2010.03.003 20451039 PMC2918406

[4] Busse WW Lemanske RF Jr Gern JE. Role of viral respiratory infections in asthma and asthma exacerbations. Lancet (2010) 376:826–34. 10.1016/S0140-6736(10)61380-3 20816549 PMC2972660

[5] Obuchi M Adachi Y Takizawa T Sata T. Influenza A(H1N1)pdm09 virus and asthma. Front Microbiol (2013) 4:307. 10.3389/fmicb.2013.00307 24133489 PMC3796256

[6] Graham AC Temple RM Obar JJ. Mast cells and influenza a virus: association with allergic responses and beyond. Front Immunol (2015) 6:238. 10.3389/fimmu.2015.00238 26042121 PMC4435071

[7] Graham AC Hilmer KM Zickovich JM Obar JJ. Inflammatory response of mast cells during influenza A virus infection is mediated by active infection and RIG-I signaling. J Immunol (2013) 190:4676–84. 10.4049/jimmunol.1202096 23526820 PMC3633673

[8] Abonia JP Hallgren J Jones T Shi T Xu Y Koni P Alpha-4 integrins and VCAM-1, but not MAdCAM-1, are essential for recruitment of mast cell progenitors to the inflamed lung. Blood (2006) 108:1588–94. 10.1182/blood-2005-12-012781 16670268 PMC1895513

[9] Hallgren J Jones TG Abonia JP Xing W Humbles A Austen KF Pulmonary CXCR2 regulates VCAM-1 and antigen-induced recruitment of mast cell progenitors. Proc Natl Acad Sci U S A (2007) 104:20478–83. 10.1073/pnas.0709651104 18077323 PMC2154456

[10] Jones TG Hallgren J Humbles A Burwell T Finkelman FD Alcaide P Antigen-induced increases in pulmonary mast cell progenitor numbers depend on IL-9 and CD1d-restricted NKT cells. J Immunol (2009) 183:5251–60. 10.4049/jimmunol.0901471 19783672 PMC2782612

[11] Collington SJ Hallgren J Pease JE Jones TG Rollins BJ Westwick J The role of the CCL2/CCR2 axis in mouse mast cell migration in vitro and in vivo. J Immunol (2010) 184:6114–23. 10.4049/jimmunol.0904177 20427772 PMC2956277

[12] Dahlin JS Feinstein R Cui Y Heyman B Hallgren J. CD11c+ cells are required for antigen-induced increase of mast cells in the lung. J Immunol (2012) 189:3869–77. 10.4049/jimmunol.1201200 22972929

[13] Bankova LG Dwyer DF Liu AY Austen KF Gurish MF. Maturation of mast cell progenitors to mucosal mast cells during allergic pulmonary inflammation in mice. Mucosal Immunol (2015) 8:596–606. 10.1038/mi.2014.91 25291985 PMC4390399

[14] Pommerenke C Wilk E Srivastava B Schulze A Novoselova N Geffers R Global transcriptome analysis in influenza-infected mouse lungs reveals the kinetics of innate and adaptive host immune responses. PLoS One (2012) 7:e41169. 10.1371/journal.pone.0041169 22815957 PMC3398930

[15] Jamur MC Grodzki AC Berenstein EH Hamawy MM Siraganian RP Oliver C Identification and characterization of undifferentiated mast cells in mouse bone marrow. Blood (2005) 105:4282–9. 10.1182/blood-2004-02-0756 15718418

[16] Chen CC Grimbaldeston MA Tsai M Weissman IL Galli SJ Identification of mast cell progenitors in adult mice. Proc Natl Acad Sci U S A (2005) 102:11408–13. 10.1073/pnas.0504197102 16006518 PMC1183570

[17] Arinobu Y Iwasaki H Gurish MF Mizuno S Shigematsu H Ozawa H Developmental checkpoints of the basophil/mast cell lineages in adult murine hematopoiesis. Proc Natl Acad Sci U S A (2005) 102:18105–10. 10.1073/pnas.0509148102 16330751 PMC1312421

[18] Dahlin JS Heyman B Hallgren J. Committed mast cell progenitors in mouse blood differ in maturity between Th1 and Th2 strains. Allergy (2013) 68:1333–7. 10.1111/all.12223 24112044 PMC4226387

[19] Dahlin JS Ding Z Hallgren J. Distinguishing mast cell progenitors from mature mast cells in mice. Stem Cells Dev (2015) 24:1703–11. 10.1089/scd.2014.0553 25744159 PMC4499794

[20] Wallach MG Webby RJ Islam F Walkden-Brown S Emmoth E Feinstein R Cross-protection of chicken immunoglobulin Y antibodies against H5N1 and H1N1 viruses passively administered in mice. Clin Vaccine Immunol (2011) 18:1083–90. 10.1128/CVI.05075-11 21613458 PMC3147324

[21] Dahlin JS Ivarsson MA Heyman B Hallgren J. IgE immune complexes stimulate an increase in lung mast cell progenitors in a mouse model of allergic airway inflammation. PLoS One (2011) 6:e20261. 10.1371/journal.pone.0020261 21625525 PMC3098291

[22] Schluter C Duchrow M Wohlenberg C Becker MH Key G Flad HD The cell proliferation-associated antigen of antibody Ki-67: a very large, ubiquitous nuclear protein with numerous repeated elements, representing a new kind of cell cycle-maintaining proteins. J Cell Biol (1993) 123:513–22. 10.1083/jcb.123.3.513 8227122 PMC2200129

[23] Starborg M Gell K Brundell E Hoog C. The murine Ki-67 cell proliferation antigen accumulates in the nucleolar and heterochromatic regions of interphase cells and at the periphery of the mitotic chromosomes in a process essential for cell cycle progression. J Cell Sci (1996) 109(Pt 1):143–53. 8834799 10.1242/jcs.109.1.143

[24] McGill J Legge KL. Cutting edge: contribution of lung-resident T cell proliferation to the overall magnitude of the antigen-specific CD8 T cell response in the lungs following murine influenza virus infection. J Immunol (2009) 183:4177–81. 10.4049/jimmunol.0901109 19767567 PMC2762786

[25] Manicassamy B Manicassamy S Belicha-Villanueva A Pisanelli G Pulendran B Garcia-Sastre A. Analysis of in vivo dynamics of influenza virus infection in mice using a GFP reporter virus. Proc Natl Acad Sci U S A (2010) 107:11531–6. 10.1073/pnas.0914994107 20534532 PMC2895123

[26] Anderson KG Mayer-Barber K Sung H Beura L James BR Taylor JJ Intravascular staining for discrimination of vascular and tissue leukocytes. Nat Protoc (2014) 9:209–22. 10.1038/nprot.2014.005 24385150 PMC4428344

[27] Yu M Tsai M Tam SY Jones C Zehnder J Galli SJ. Mast cells can promote the development of multiple features of chronic asthma in mice. J Clin Invest (2006) 116:1633–41. 10.1172/JCI25702 16710480 PMC1462940

[28] Waern I Jonasson S Hjoberg J Bucht A Abrink M Pejler G Mouse mast cell protease 4 is the major chymase in murine airways and has a protective role in allergic airway inflammation. J Immunol (2009) 183:6369–76. 10.4049/jimmunol.0900180 19841188

[29] Ajami B Bennett JL Krieger C Tetzlaff W Rossi FM. Local self-renewal can sustain CNS microglia maintenance and function throughout adult life. Nat Neurosci (2007) 10:1538–43. 10.1038/nn2014 18026097

[30] Law MP. Vascular permeability and late radiation fibrosis in mouse lung. Radiat Res (1985) 103:60–76. 10.2307/3576671 4070560

[31] Lei Y Gregory JA Nilsson GP Adner M. Insights into mast cell functions in asthma using mouse models. Pulm Pharmacol Ther (2013) 26:532–9. 10.1016/j.pupt.2013.03.019 23583635

[32] Janssen WJ Barthel L Muldrow A Oberley-Deegan RE Kearns MT Jakubzick C Fas determines differential fates of resident and recruited macrophages during resolution of acute lung injury. Am J Respir Crit Care Med (2011) 184:547–60. 10.1164/rccm.201011-1891OC 21471090 PMC3175550

[33] Nocka K Tan JC Chiu E Chu TY Ray P Traktman P Molecular bases of dominant negative and loss of function mutations at the murine c-kit/white spotting locus: W37, Wv, W41 and W. EMBO J (1990) 9:1805–13. 1693331 10.1002/j.1460-2075.1990.tb08305.x PMC551885

[34] Grimbaldeston MA Chen CC Piliponsky AM Tsai M Tam SY Galli SJ. Mast cell-deficient W-sash c-kit mutant Kit W-sh/W-sh mice as a model for investigating mast cell biology in vivo. Am J Pathol (2005) 167:835–48. 10.1016/S0002-9440(10)62055-X 16127161 PMC1698741

[35] Zsebo KM Williams DA Geissler EN Broudy VC Martin FH Atkins HL Stem cell factor is encoded at the Sl locus of the mouse and is the ligand for the c-kit tyrosine kinase receptor. Cell (1990) 63:213–24. 10.1016/0092-8674(90)90302-U 1698556

[36] Moller C Alfredsson J Engstrom M Wootz H Xiang Z Lennartsson J Stem cell factor promotes mast cell survival via inactivation of FOXO3a-mediated transcriptional induction and MEK-regulated phosphorylation of the proapoptotic protein Bim. Blood (2005) 106:1330–6. 10.1182/blood-2004-12-4792 15855272

[37] Miura M Inoue H Ichinose M Shimura S Katsumata U Kimura K Increase in luminal mast cell and epithelial damage may account for increased airway responsiveness after viral infection in dogs. Am Rev Respir Dis (1989) 140:1738–44. 10.1164/ajrccm/140.6.1738 2604300

[38] Sorden SD Castleman WL. Brown Norway rats are high responders to bronchiolitis, pneumonia, and bronchiolar mastocytosis induced by parainfluenza virus. Exp Lung Res (1991) 17:1025–45. 10.3109/01902149109064333 1663031

[39] Alho OP Karttunen TJ Karttunen R Tuokko H Koskela M Uhari M. Lymphocyte and mast cell counts are increased in the nasal mucosa in symptomatic natural colds. Clin Exp Immunol (2003) 131:138–42. 10.1046/j.1365-2249.2003.02037.x 12519397 PMC1808600

[40] Balzar S Fajt ML Comhair SA Erzurum SC Bleecker E Busse WW Mast cell phenotype, location, and activation in severe asthma. Data from the Severe Asthma Research Program. Am J Respir Crit Care Med (2011) 183:299–309. 10.1164/rccm.201002-0295OC 20813890 PMC3056228

[41] Dahlin JS Malinovschi A Ohrvik H Sandelin M Janson C Alving K Lin- CD34hi CD117int/hi FcepsilonRI+ cells in human blood constitute a rare population of mast cell progenitors. Blood (2016) 127:383–91. 10.1182/blood-2015-06-650648 26626992 PMC4731844

