# Supplementary material for: Influenza Infection in Mice Induces Accumulation of Lung Mast Cells through the Recruitment and Maturation of Mast Cell Progenitors
Source: Front Immunol. 2017 Mar 22;8:310. doi: 10.3389/fimmu.2017.00310 (PMC5360735; doi:10.3389/fimmu.2017.00310)
Supplement: Supplementary file 1 [file Presentation_1.pdf]

Supplementary Material

Influenza infection in mice induces accumulation of lung mast cells through the recruitment and maturation of mast cell progenitors

B. Zarnegar[1], E. Mendez-Enriquez[1], A. Westin[1], C. Söderberg[1], J. S. Dahlin[1,2], K-O. Grönvik[1,3] and J. Hallgren[1*]

[1] Department of Medical Biochemistry and Microbiology, BMC, Uppsala University, Uppsala, Sweden.

[2] Present address: Department of Medicine, Karolinska Institutet, Karolinska University Hospital, Stockholm, Sweden.

[3] Uppsala Immunobiology Lab, c/o National Veterinary Institute, Uppsala, Sweden.

*** Correspondence:** Jenny Hallgren Martinsson: jenny.hallgren@imbim.uu.se

Supplementary Figures

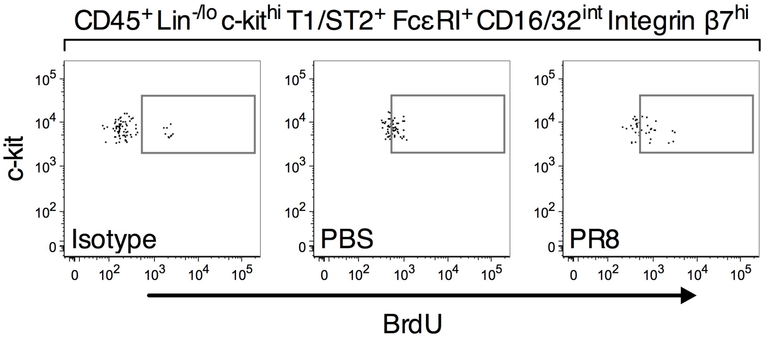

Supplementary Figure 1 (S1). A high proportion of MCp-like cells in the bone marrow are positive for BrdU. Wild type mice were infected with PR8 influenza virus or received PBS intranasally. On day seven post-infection, all mice were given BrdU intranasally. The next day, bone marrow cells were isolated from femur and tibia by flushing the bones using PBS and treated with red blood cell lysis buffer. To detect $BrdU^+$ MCp-like cells, the bone marrow cells were stained with fluorescently labeled antibodies, fixated, and permeabilized before staining with anti-BrdU antibodies and analyzed by flow cytometry. The figure displays representative dot plots of $CD45^+$ $Lin^{-/lo}$ c-kit^{hi} $T1/ST2^+$ $Fc\varepsilon RI^+$ $CD16/32^{int}$ integrin $\beta7^{hi}$ cells day eight post-infection from nine mice per group pooled from two individual experiments. The flow cytometry plot labeled as isotype shows a FMO control with an appropriate mouse IgG1 isotype antibody added. The gate illustrates $BrdU^+$ cells.

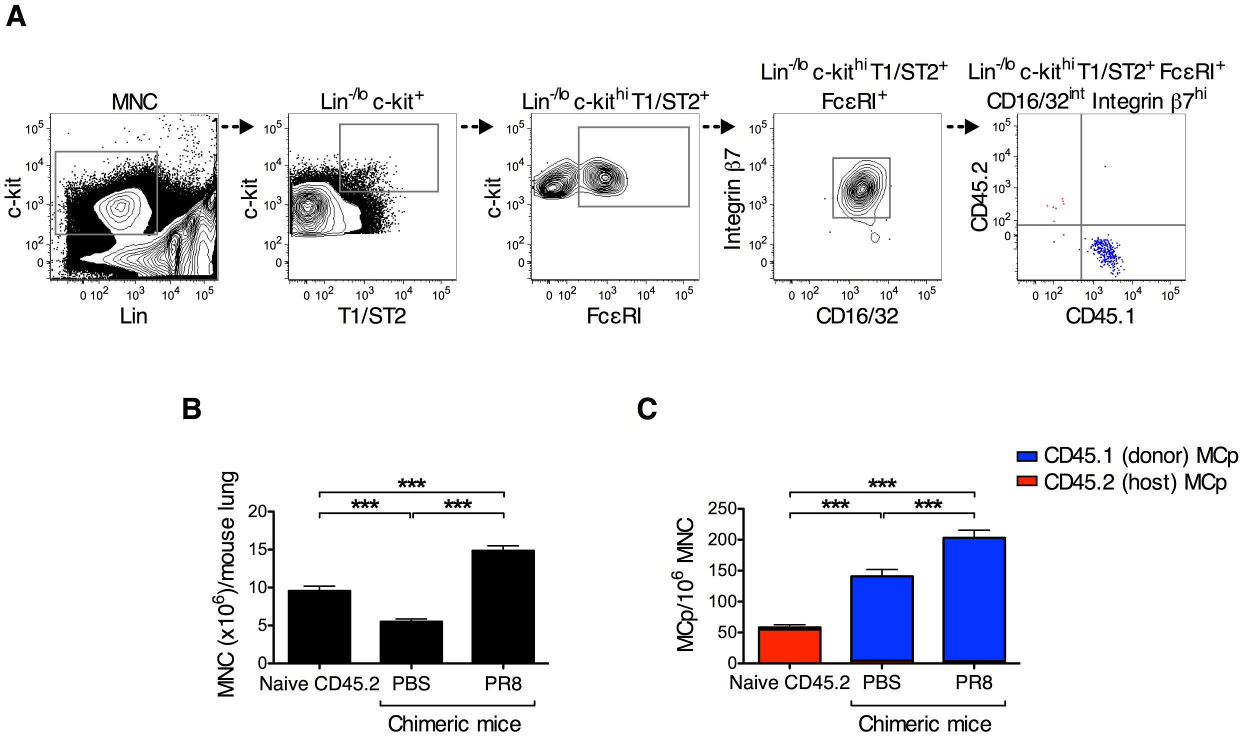

Supplementary Figure 2 (S2). Influenza infection stimulates the recruitment of mast cell progenitors to the lung. CD45.2 BALB/c mice were sublethally γ-irradiated with 5 Gy, and reconstituted with bone marrow from CD45.1 BALB/c mice, two days before infection. Eight days after PR8 infection or PBS treatment, the mice were euthanized and analyzed for the presence of $CD45.1^+$ and $CD45.2^+$ MCp using the gating strategy depicted in **(A)**. The sample shown is from a PR8-infected chimeric mouse eight days post-infection. **(B)** The total number of lung MNC and (**C**) the frequency of MCp/10^6 MNC eight days post-infection or in naïve CD45.2 mice. **(C)** The $CD45.1^+$ MCp are represented in blue while the $CD45.2^+$ MCp are represented in red. The graphs in **(B)** and **(C)** show the mean ± SEM from 12-15 individual mice per group pooled from three independent experiments. All groups were compared to each other and therefore the statistical significance was determined by one-way ANOVA with post-hoc Tukey's test. *** $P < 0.001$.
